# p75NTR promotes tooth rhythmic mineralization via upregulation of BMAL1/CLOCK

**DOI:** 10.3389/fcell.2023.1283878

**Published:** 2023-11-07

**Authors:** Bo Xie, Hongyan Yuan, Xuqiang Zou, Mingjie Lu, Yixin Zhang, Dan Xu, Xuelian Peng, Di Wang, Manzhu Zhao, Xiujie Wen

**Affiliations:** ^1^ Department of Orthodontics, School of Stomatology, Southwest Medical University, Luzhou, China; ^2^ Chongqing Key Laboratory for Oral Diseases and Biomedical Sciences, Chongqing Municipal Key Laboratory of Oral Biomedical Engineering of Higher Education, College of Stomatology, Chongqing Medical University, Chongqing, China

**Keywords:** p75NTR, ectomesenchymal stem cells, circadian rhythm, mineralization, odontogenic differentiation, tooth development

## Abstract

The circadian clock plays a critical role in dentomaxillofacial development. Tooth biomineralization is characterized by the circadian clock; however, the mechanisms underlying the coordination of circadian rhythms with tooth development and biomineralization remain unclear. The p75 neurotrophin receptor (p75NTR) is a clock factor that regulates the oscillatory components of the circadian rhythm. This study aims to investigate the impact of p75NTR on the rhythmic mineralization of teeth and elucidate its underlying molecular mechanisms. We generated p75NTR knockout mice to examine the effects of p75NTR deficiency on tooth mineralization. Ectomesenchymal stem cells (EMSCs), derived from mouse tooth germs, were used for *in vitro* experiments. Results showed a reduction in tooth mineral density and daily mineralization rate in p75NTR knockout mice. Deletion of p75NTR decreased the expression of DMP1, DSPP, RUNX2, and ALP in tooth germ. Odontogenic differentiation and mineralization of EMSCs were activated by p75NTR. Histological results demonstrated predominant detection of p75NTR protein in odontoblasts and stratum intermedium cells during rapid formation phases of dental hard tissue. The mRNA expression of *p75NTR* exhibited circadian variations in tooth germs and EMSCs, consistent with the expression patterns of the core clock genes *Bmal1* and *Clock*. The upregulation of BMAL1/CLOCK expression by p75NTR positively regulated the mineralization ability of EMSCs, whereas BMAL1 and CLOCK exerted a negative feedback regulation on p75NTR by inhibiting its promoter activity. Our findings suggest that p75NTR is necessary to maintain normal tooth biomineralization. Odontogenic differentiation and mineralization of EMSCs is regulated by the p75NTR-BMAL1/CLOCK signaling axis. These findings offer valuable insights into the associations between circadian rhythms, tooth development, and biomineralization.

## 1 Introduction

The circadian clock is an evolutionarily conserved timekeeping mechanism that regulates most physiological processes, including hormone secretion, metabolism, growth, and sleep (Green et al., 2008a; Hasler et al., 2012a; Neumann et al., 2019a). The suprachiasmatic nucleus (SCN), located in the anterior hypothalamus of the brain, is the master clock for the regulation of the circadian rhythm (Abel et al., 2016). External light signals are converted into biological signals by the SCN and transmitted to the peripheral clocks of various organs and tissues through neurons or endocrine pathways, thereby forming a highly unified and coherent circadian rhythm network that enables the body to adapt to 24-h diurnal variation (Bell-Pedersen et al., 2005; Mohawk et al., 2012a; Buijs et al., 2016a). At the molecular level, the regulation of clock rhythm depends on the transcription-translation feedback loop (TTFL) composed of clock genes (Duong et al., 2011a). The transcription of clock-controlled genes, including period (*Per*) and cryptochrome (*Cry*) genes, is activated by the BMAL1/CLOCK heterodimer formed by the core clock factors BMAL1 and CLOCK (Gekakis et al., 1998; Reppert and Weaver, 2001a). Upon reaching a critical level, the PER and CRY proteins are phosphorylated and transferred to the nucleus to inhibit the activation of BMAL1/CLOCK, thereby downregulating their expression (Brown et al., 2012a).

Rhythmic characteristics have been observed in dental hard tissues. The cross striations and Retzius lines in the enamel and von Ebner incremental lines in the dentin are traces left by the circadian clock during tooth biomineralization (Ohtsuka et al., 1998a; Smith, 2006a). The formation of incremental lines in dentin is disturbed in rats with damaged SCN (Ohtsuka-Isoya et al., 2001a). In ameloblasts, the expression of clock genes showed rhythmicity (Zheng et al., 2011a; Lacruz et al., 2012). Collagen synthesis and secretion exhibit circadian rhythms in odontoblasts (Ohtsuka et al., 1998a). Although the circadian clock plays an important role in the regulation of tooth development and mineralization, the underlying molecular mechanisms remain unclear.

The biological roles of clock genes in tooth development have attracted attention. p75 neurotrophin receptor (p75NTR), a member of the tumor necrosis factor superfamily, is a novel clock factor capable of regulating tooth development through the activation of multiple signaling pathways including the TGF-β/SMAD, PI3K/Akt/β-Catenin, and Wnt/β-Catenin pathways (Xing et al., 2016; Li et al., 2017; Wang et al., 2020). Absence of p75NTR significantly reduces the daily mineralization rate of mouse incisors (Zhao et al., 2020). Interestingly, in rat ectomesenchymal stem cells (EMSCs) derived from tooth germ (primordium of the tooth), *p75NTR* mRNA showed circadian variation, consistent with the expression patterns of the core clock genes *Bmal1* and *Clock* (Yuan et al., 2022). This suggests that p75NTR acts as a clock factor to coordinate tooth development and biomineralization. Therefore, the present study focused on investigating the p75NTR-mediated regulation of tooth rhythmic mineralization and odontogenic differentiation of EMSCs.

## 2 Materials and methods

### 2.1 Animals

p75NTR knockout (KO; p75NTR−/−) mice (C57BL/6) were generated by Cyagen Bioscience, Inc (Guangzhou, China). Heterozygous mice (p75NTR+/−) were interbred to obtain wildtype (WT; p75NTR+/+) and homozygous null littermates (p75NTR−/−). Genotyping of WT and KO mice was performed with the following primers: forward primer 5ʹ-CCT​GGT​AGT​AAG​CAG​CTC​AAT​G-3ʹ and reverse primer 5ʹ-TAA​AAA​TAC​CAC​CGA​GCA​CAA​GGC-3ʹ for p75NTR KO mice; forward primer 5ʹ-CCT​GGT​AGT​AAG​CAG​CTC​AAT​G-3ʹ and reverse primer 5ʹ-ATA​GAA​ACA​AGG​GAC​AGA​CCA​AGA​G-3ʹ for WT mice.

Postnatal day 1 (PN1d), PN7d, and PN14d WT C57BL/6 mice were purchased from Southwest Medical University Laboratory Animal Center (Luzhou, China). Tooth germs were dissected from the alveolar fossa of PN1d mice for EMSCs culture or extracting mRNA and protein for subsequent experiments. The mandibles were dissected from PN7d and PN14d mice and used to generate paraffin sections for immunofluorescence staining.

All mice were kept under standard laboratory conditions at the Southwest Medical University Laboratory Animal Center. All animal experiments were approved by the Committee on the Ethics of Animal Experiments of Southwest Medical University (authorization number: 201903-187).

### 2.2 Micro-computed tomography (micro-CT) analysis

Mandibles were dissected from eight-week-old male WT or p75NTR KO mice and fixed in 4% paraformaldehyde (PFA) for 24 h. A micro-CT (Viva CT 40; Scanco Medical AG, Switzerland) was used to scan the samples. MicroView software version 2.2 (GE Health Systems, United States) was used to calculate the three-dimensional (3D) microstructural parameters and analyze the tissue surface area, percent volume, and mineral density of the region of interest (ROI; the crown of the mandibular first molar).

### 2.3 Analysis of incisor eruption speed

The incisor eruption speed was determined as previously described (Huang et al., 2021a). Eight-week-old male WT and p75NTR KO mice were used in this experiment. A high-speed dental handpiece was used to generate small dimples on the labial enamel surface of the gingival margins. One week later, the distance between the small dimples and the gingival margin was determined and designated as the length of the incisor eruption.

### 2.4 Calcein fluorescence assay

Neonatal p75NTR KO and WT littermates were injected intraperitoneally with calcein (25 mg/kg) at 8:00 a.m. on PN7d and every 7 days thereafter until a total of four injections had been administered. The mice were euthanized by CO_2_ inhalation 3 days after the last injection. The mandibles were dissected from the mice and fixed in 4% PFA for 24 h. After embedding, we generated 8-μm-thick sections using an EXAKT precision cutting and grinding system (EXAKT Vertriebs GmbH). Calcein fluorescence images were obtained using a fluorescence microscope (Olympus, Japan).

### 2.5 Isolation, culture, and identification of EMSCs

To obtain EMSCs, tooth germs were dissected from the mandibles of PN1d WT C57BL/6 mice. Then, tooth germ tissues were digested with collagenase type I (10 μg/mL) at 37 °C for 15 min. After centrifugation (1,000 rpm, 5 min), the supernatant was discarded. Subsequently, Dulbecco’s Modified Eagle Medium (DMEM)/F12 medium supplemented with 10% fetal bovine serum (FBS) and 1% antibiotic solution (100 μg/mL penicillin and 100 μg/mL streptomycin) was added to culture the EMSCs at 37 °C in a 5% CO_2_ humidified incubator.

Flow cytometry (FACSCalibur, BD Biosciences, CA, United States) was performed to detect EMSCs surface markers. Third-passage EMSCs were digested with 0.25% trypsin-ethylenediaminetetraacetic acid. The cell pellet was resuspended in phosphate-buffered saline (PBS). The EMSCs were incubated at 4 °C with mouse antibodies to detect CD29 (APC-labeled), CD34 (Alexa Fluor 647-labeled), CD45 (APC-labeled), CD90 (APC-labeled), and CD105 (APC-labeled) (Bio-legend, CA, United States). Anti-p75NTR (FITC-labeled) (Santa Cruz, CA, United States) was used to detect p75NTR. Subsequently, flow cytometry was performed to detect these markers.

### 2.6 Lentiviral transductions and small interfering RNA (siRNA) transfections

Lentiviral vectors encoding p75NTR, BMAL1, or CLOCK were generated using the GV492 vector (GeneChem, Shanghai, China) and designated as OE-p75NTR, OE-BMAL1, and OE-CLOCK, respectively. An empty vector was used as a negative control (OE-NC). Following the manufacturer’s instructions, lentiviral transduction was performed to generate EMSCs overexpressing p75NTR, BMAL1, or CLOCK.

Based on the overexpression of p75NTR, we constructed BMAL1/CLOCK-knockdown EMSCs using BMAL1 and CLOCK siRNA and designated them as OE-p75NTR + si-BMAL1/CLOCK. siRNAs (BMAL1-and CLOCK-targeted or negative control siRNAs) were obtained from Santa Cruz Biotechnology (Santa Cruz, CA, USA). Briefly, EMSCs overexpressing p75NTR were cultured in six-well plates to the appropriate confluence and transfected with siRNAs (2 μg/well) using Lipofectamine 2000 (Invitrogen, CA, United States) for 24 h. The medium was removed and replaced with a complete DMEM/F12 medium for subsequent experiments.

### 2.7 Immunofluorescence staining

p75NTR overexpression EMSCs and negative control EMSCs were plated evenly on slides in a complete DMEM/F12 medium. After the medium was removed, the EMSCs were washed with PBS, fixed with 4% PFA, and permeabilized with 0.5% Triton X-100. We used 5% bovine serum albumin to block nonspecific antibody-binding sites. The cells were then incubated with primary antibodies against p75NTR (1:100, ab52987, Abcam), BMAL1 (1:100, DF10308, Affinity), or CLOCK (1:100, ab3517, Abcam) at 4 °C overnight. The following day, the cells were washed and incubated with goat anti-rabbit secondary antibodies (1:200, Beyotime) for 1 h. For the tooth sections, primary antibodies against p75NTR (1:200, ab52987, Abcam) were applied, followed by incubation with Alexa Fluor 594 goat anti-rabbit secondary antibodies (1:1,000, Invitrogen). DAPI (Beyotime) was used to stain the nuclei. Images were captured using a fluorescence microscope (Olympus).

### 2.8 Mineralization induction of EMSCs, alkaline phosphatase (ALP) staining, and alizarin red staining

After lentiviral transduction or siRNA transfection, the medium was replaced with a mineralization induction medium (containing 50 mg/mL ascorbic acid, 10 mmol/L β-glycerophosphate, and 10 μmol/L dexamethasone). The mineralization induction medium was changed every 3 days. Seven days after induction, the cells were fixed with 4% PFA for 15 min. An ALP assay kit (Beyotime) was used to detect ALP activity following the manufacturer’s instructions. At 21 days post-induction, we performed alizarin red staining (Cyagen, Guangzhou, China) following the manufacturer’s protocol to detect the formation of calcium nodules.

### 2.9 Circadian rhythm induction of EMSCs

EMSCs were synchronized as described in a previous study (Tang et al., 2019). Briefly, when third-passage EMSCs reached 100% confluence, the medium was replaced with fresh medium containing 1 μmol/L dexamethasone (Sigma). After 2 h, the medium was replaced with a fresh complete medium. This time point was defined as circadian time 0 (CT0). After 4 h, the time point was defined as CT4.

### 2.10 Quantitative reverse transcription (qRT)-PCR analysis

TRIzol reagent (Takara, Tokyo, Japan) was used to obtain total RNA from the tissues or EMSCs following the manufacturer’s instructions. cDNA was generated by reverse transcription using the PrimeScript Reverse Transcription Reagent Kit (Takara). qPCR was performed to detect the expression levels of *Bmal1*, *Clock*, *p75NTR*, *Per1*, *Per2*, and *Cry1* using the SYBR Premix ExTaq kit (Takara). The primer sequences are listed in Table 1. Relative mRNA expression was normalized to that of Gapdh using the 2^-△△Ct^ method.

**TABLE 1:** Primer sequences used in this study.

mRNA name	Forward primers (5′–3′)	Reverse primers (5′–3′)	Product size (bp)
*Gapdh*	TGA​CCT​CAA​CTA​CAT​GGT​CTA​CA	CTT​CCC​ATT​CTC​GGC​CTT​G	85
*p75NTR*	CCA​GAG​CGA​GAC​CTC​ATA​GC	AGA​TGG​AGC​AAT​AGA​CAG​GAA​TG	121
*Bmal1*	AAG​ACA​ATG​AGC​CAG​ACA​ACG	TCC​CAT​CTA​TTG​CGT​GTC​G	147
*Clock*	CCA​TCC​AGT​ATG​CCA​CAG​AAC	TCA​CCA​CCT​GAC​CCA​TAA​GC	172
*Per1*	CCA​GTA​CAA​CCA​AGC​GTA​AAT​G	TTG​CTG​ACG​ACG​GAT​CTT​TC	123
*Per2*	AGC​GGC​TTA​GAT​TCT​TTC​ACT​C	TCT​CAT​TCT​CGT​GGT​GTT​TCC	88
*Cry1*	ACT​CCC​GTC​TGT​TTG​TGA​TTC​G	GCT​GCG​TCT​CGT​TCC​TTT​CC	127

### 2.11 Western blotting

Total protein from tooth germ tissues and EMSCs was extracted using RIPA buffer (Beyotime, Shanghai, China) containing 1 mM phenylmethanesulfonyl fluoride (PMSF). The concentrations of all protein samples were determined using a BCA protein assay kit (Beyotime). Equal quantities of protein were separated using sodium dodecyl sulfate-polyacrylamide gel electrophoresis (SDS-PAGE) and transferred onto polyvinylidene difluoride (PVDF) membranes. After blocking, the membranes were incubated at 4 °C overnight with antibodies against BMAL1 (1:1,000, ab230822, Abcam), CLOCK (1:1,000, ab3517, Abcam), p75NTR (1:1,000, ab52987, Abcam), DMP1 (1:200, sc-73633, Santa Cruz Biotechnology), DSPP (1:200, sc-73632, Santa Cruz Biotechnology), RUNX2 (1:1,000, ab236639, Abcam), ALP (1:200, sc-365765, Santa Cruz Biotechnology), or β-ACTIN (1:5,000, AF7018, Affinity Biosciences). The following day, the membranes were washed and incubated with the corresponding horseradish peroxidase-conjugated secondary antibodies (Proteintech, Wuhan, China). Reactive signals were detected using ECL (Thermo Fisher Scientific, MA, United States) and exposed to autoradiography film (Amersham, United Kingdom).

### 2.12 Luciferase reporter assay

BMAL1 expression plasmid, CLOCK expression plasmid, firefly luciferase reporter plasmid containing the *p75NTR* promoter, and the corresponding negative control plasmid were purchased from GeneChem (Shanghai, China). HEK293T cells (GeneChem) were seeded in 24-well plates and cultured until they reached 60% confluence. Each well was transfected with 0.5 μg of firefly luciferase reporter plasmid, 0.5 μg of BMAL1 and/or CLOCK expression plasmid, and 0.02 μg of Renilla luciferase expression plasmid (Ambion, United States) using the X-tremeGENE HP DNA Transfection Reagent (Roche, Basel, Switzerland). After 48 h, the Dual-Luciferase^®^ Reporter Assay System (Promega, WI, United States) was used to detect luciferase activity, according to the manufacturer’s instructions. Negative control groups were also established to confirm the effectiveness of the system.

### 2.13 Statistical analysis

Each quantitative experiment was performed in triplicates. Data analysis and graphing were performed using GraphPad Prism software (version 9.0; GraphPad Software, San Diego, CA, United States). The results are presented as mean ± standard deviation (SD). Differences between groups were analyzed using unpaired two-tailed Student's *t*-tests with a *p*-value <0.05 indicating statistical significance.

## 3 Results

### 3.1 p75NTR-KO mice show decreased tooth mineral density and daily mineralization rate

**FIGURE 1:**
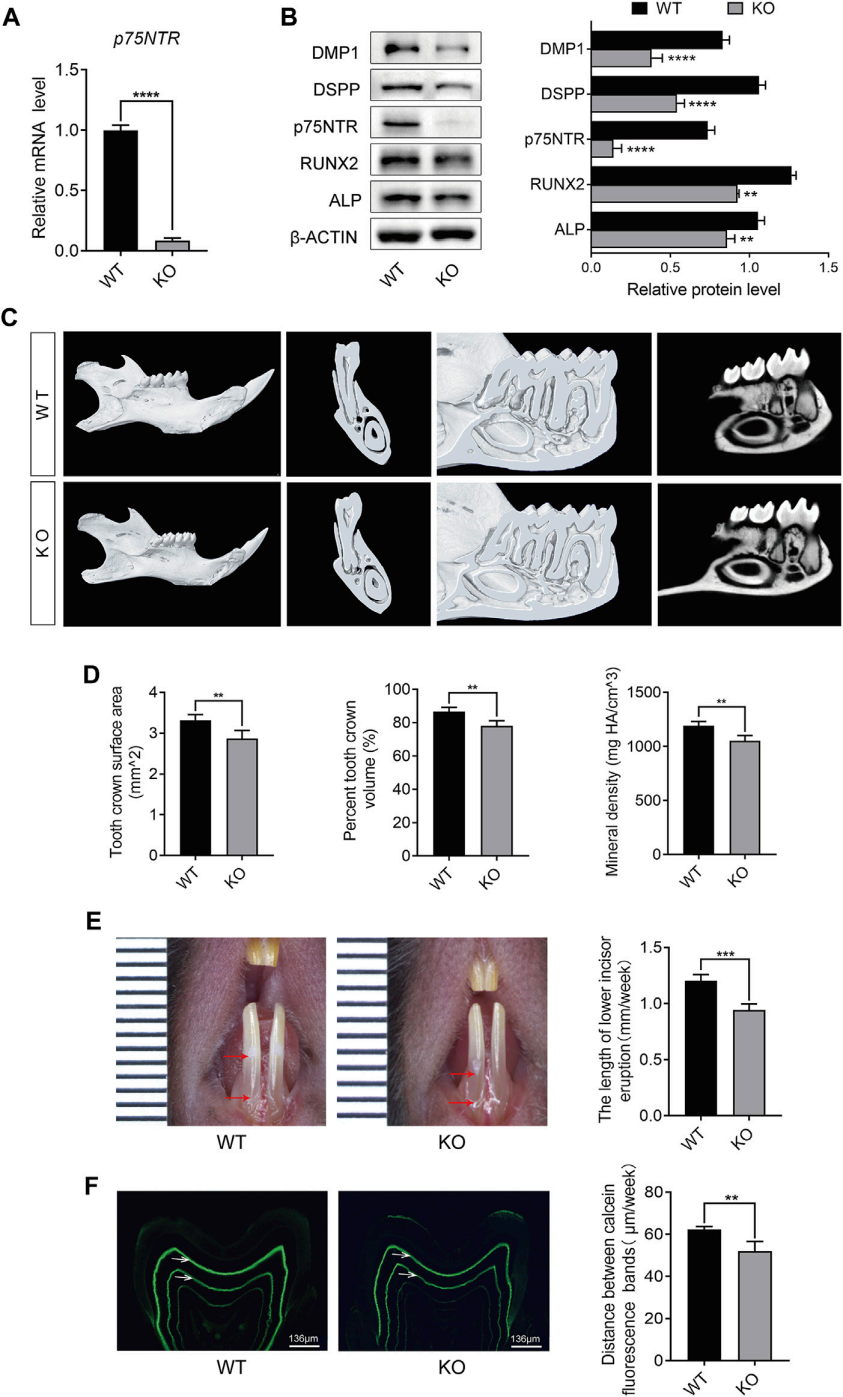
p75NTR knockout (KO) mice showed decreased tooth mineral density and daily mineralization rate. **(A)** Quantitative reverse transcription (qRT)-PCR analysis of p75NTR in tooth germ tissues of wildtype (WT) and knockout (KO) mice. **(B)** Western blot and quantification analysis of p75NTR, DMP1, DSPP, RUNX2, and ALP in tooth germ from WT and KO mice (n = 3). **(C)** Representative micro-computed tomography (micro-CT) images of the mandibles collected from 8-week-old male WT and KO mice. **(D)** Quantitative analysis of the surface area, percent volume, and mineral density of the first mandibular molar crowns from micro-CT (n = 5). **(E)** Quantitative analysis of incisor eruption speed. The length between the top and bottom red arrows indicates the amount of lower incisor eruption for 1 week (n = 5). **(F)** Representative images of a mandibular first molar of WT and KO mice labeled by calcein and quantitative analysis of mineralization rate (n = 5). The distance between the bands indicated by white arrows represents the cumulative mineralization for 1 week. Scale bar = 136 μm. Data are shown as the mean ± standard deviation. **p ≤ 0.01, ***p ≤ 0.005, and ****p ≤ 0.0001.

To clarify the physiological function of p75NTR *in vivo*, we generated p75NTR KO mice on a C57BL/6 background. The gene-targeting strategy is illustrated in Supplementary Figure S1. qRT-PCR analysis showed that the expression of *p75NTR* in the tooth germ of KO mice was decreased by approximately 90% compared to that in WT mice (Figure 1A). DMP1, DSPP, RUNX2, and ALP are markers of odontogenic differentiation and mineralization (Chapple, 1993a; D'Souza et al., 1999; Ritchie, 2018a; Ye et al., 2004). Western blot analysis demonstrated that, with a significant decrease in p75NTR, the expression of DMP1, DSPP, RUNX2, and ALP decreased significantly (Figure 1B). Furthermore, the surface area and volume fraction of the mandibular first molar crown decreased in KO mice, as determined via micro-CT analysis (Figures 1C, D). The tooth mineral density of p75NTR KO mice (1.05 g HA/cm^3^) was significantly lower than that of the WT mice (1.19 g HA/cm^3^) (Figure 1D).

By marking the surface of the mandibular incisors, we found that the average daily eruption length of the incisors in the KO mice decreased by approximately 38 μm (Figure 1E). In addition, the tooth mineral processes of WT and KO mice were labeled with calcein (Figure 1F). Fluorescence analysis revealed that calcein only marked the dentin, and there was no marked line in the enamel. Compared with WT mice, KO mice showed shorter distances between labeled lines (indicated by white arrows), which were approximately 10.3 μm shorter (Figure 1F). Notably, the marked lines of KO mice showed lower fluorescence intensity. These data suggest that p75NTR deletion reduces tooth mineral density and daily mineralization rate.

### 3.2 Tissue-specific expression of p75NTR protein during tooth development

**FIGURE 2:**
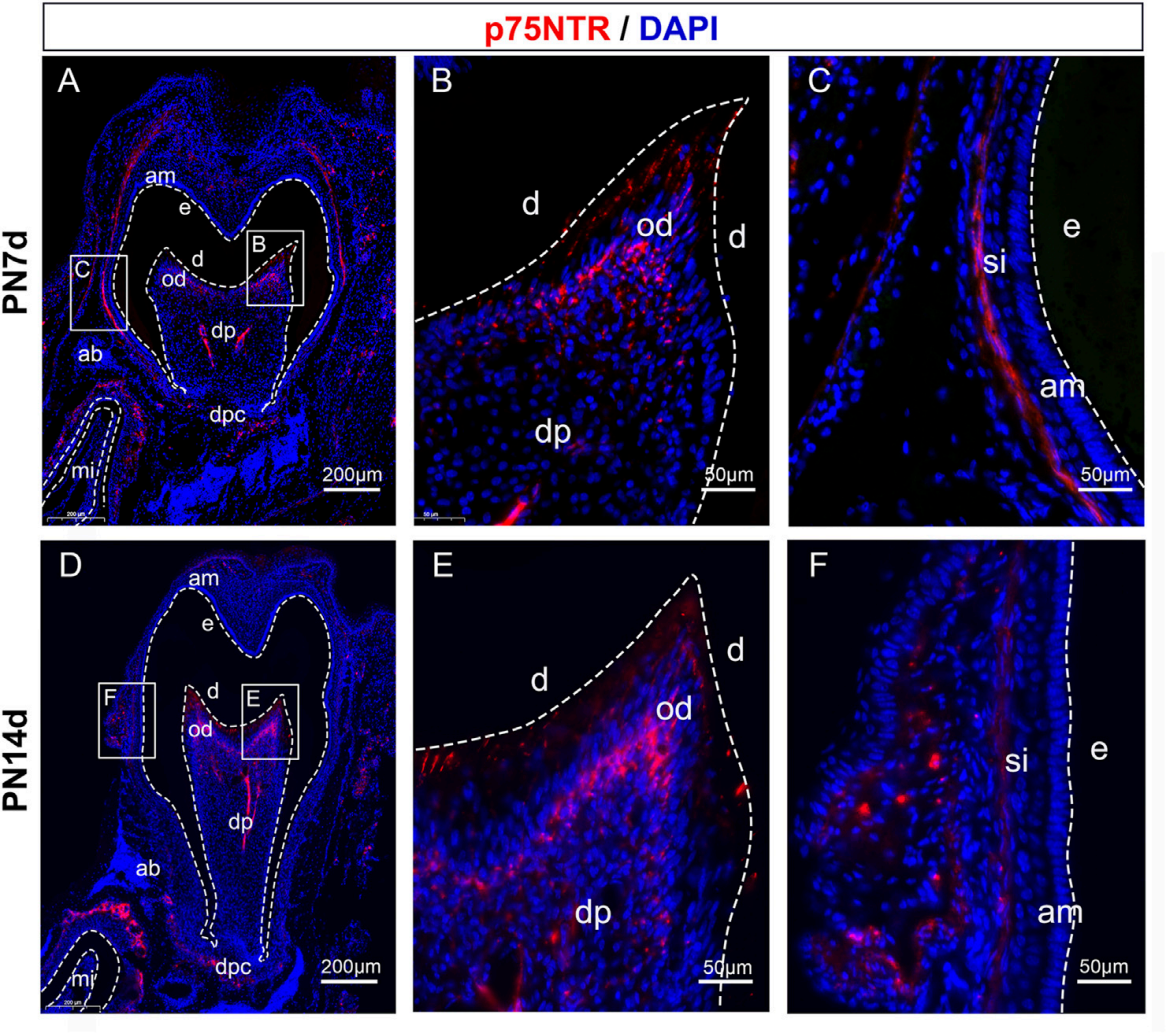
Tissue-specific expression of p75NTR protein during tooth development. Immunofluorescence staining of p75NTR (red) in mandibular first molar tissues from **(A-C)** PN7d and **(D-F)** PN14d wildtype mice. The boxed areas are magnified in the right panels. Nuclear staining was performed with DAPI dye (blue). Scale bar = 200 or 50 μm. am, ameloblast; e, enamel; d, dentin; od, odontoblast; dp, dental pulp; dpc, dental papilla cells; ab, alveolar bone; mi, mandibular incisor; si, stratum intermedium cells; PN7d, postnatal day 7; PN14d, postnatal day 14.

During early odontogenesis, p75NTR is highly expressed in the epithelial-mesenchymal interaction area (Xing et al., 2016; Zhao et al., 2019; Yuan et al., 2022). Epithelial and mesenchymal cells differentiate into ameloblasts and odontoblasts, respectively, and participate in the formation of dental hard tissues (including enamel and dentin) (Yu and Klein, 2020a). Immunofluorescence staining demonstrated the tissue-specific expression of p75NTR during the rapid formation phase of dental hard tissue (Figures 2A–F). p75NTR is mainly expressed in odontoblasts at the top of the tooth pulp and in stratum intermedium cells outside the ameloblasts. Low levels of expression were also observed in the apical region of the molars and around the roots of the incisors.

### 3.3 p75NTR positively regulates odontogenic differentiation of EMSCs

**FIGURE 3:**
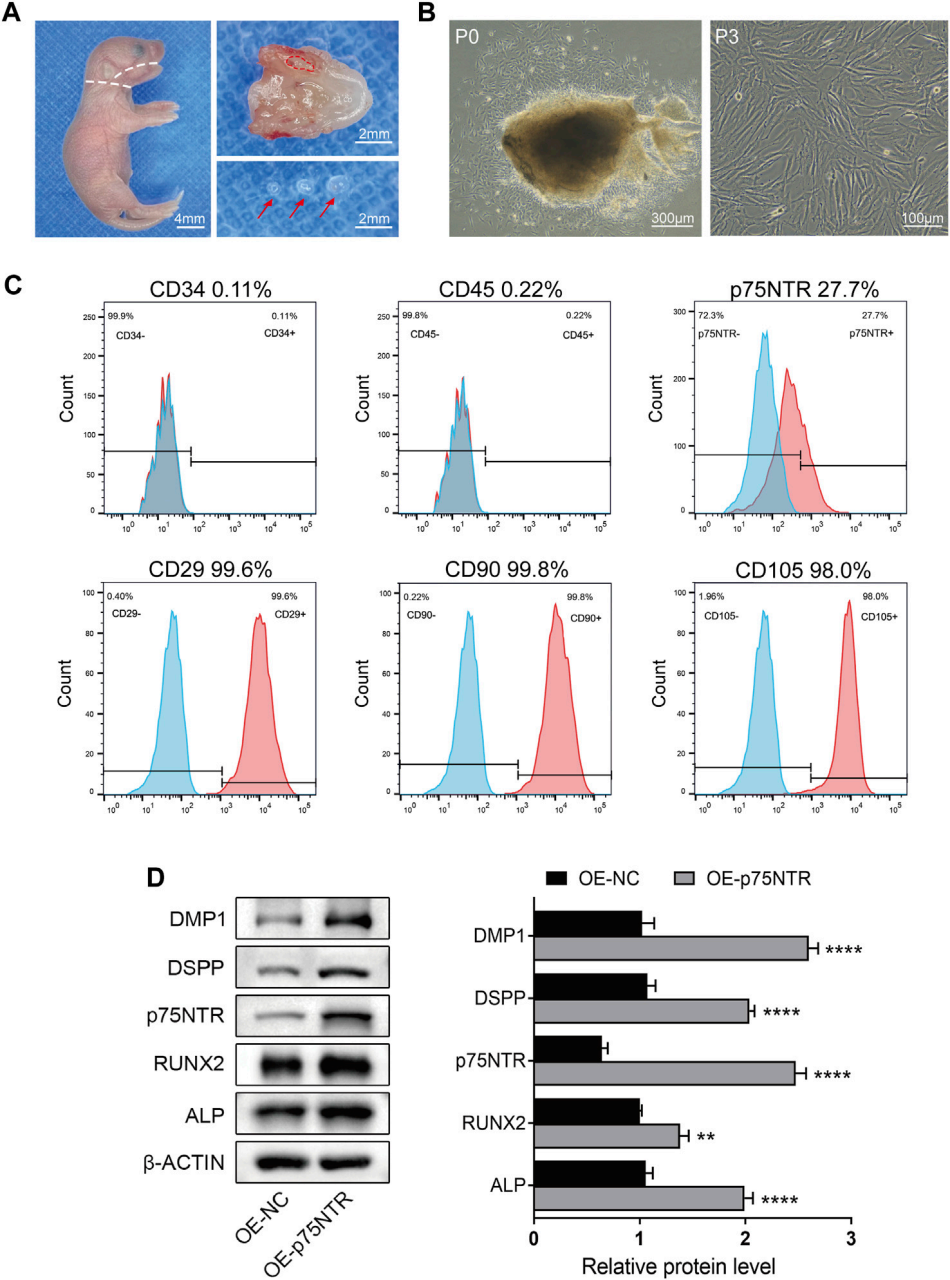
p75NTR positively regulated odontogenic differentiation of ectomesenchymal stem cells (EMSCs). **(A)** Tooth germ tissues were dissected from the mandibles of postnatal day 1 wildtype mice. The mandibles were separated along the white dotted line, and tooth germ was dissected from the alveolar fossa (red dotted line area). Red arrows indicate complete tooth germs. Scale bar = 4 or 2 mm. **(B)** Morphology of primary (P0) and third-passage EMSCs (P3) was observed using light microscopy. Scale bar = 300 or 100 μm. **(C)** Flow cytometry was performed to identify cell surface markers (CD29, CD34, CD45, CD90, CD105, and p75NTR). The *x*-axis and *y*-axis represent the fluorescence signal intensity and the number of cells, respectively. Red color represents positive cells, while blue color represents negative cells. **(D)** Representative western blots and quantification of the indicated proteins in p75NTR overexpressing (OE-p75NTR) and negative control (OE-NC) EMSCs (n = 3). Data are shown as mean ± standard deviation. **p ≤ 0.01 and ****p ≤ 0.0001 as indicated.

To explore the mechanism underlying the effect of p75NTR on tooth mineralization, EMSCs were cultured from the tooth germs of WT mice. The cells showed uniform fibroblast-like morphology and strong growth activity (Figures 3A, B). Flow cytometry was used to detect the surface markers of stem cells and the expression of p75NTR (Figure 3C). The results showed that CD29 (99.6%), CD90 (99.8%), and CD105 (98.0%) were highly expressed, and the expression rate of p75NTR was 27.7%. The hematopoietic stem cell markers CD45 (0.22%) and CD34 (0.11%) were barely detectable (Figure 3C). EMSCs were then transduced with a lentivirus to overexpress p75NTR (OE-p75NTR). Compared with the negative control EMSCs (OE-NC), overexpression of p75NTR promoted the expression of proteins such as DMP1, DSPP, RUNX2, and ALP (Figure 3D). This indicates that p75NTR promotes the odontogenic differentiation of EMSCs.

### 3.4 p75NTR, BMAL1, and CLOCK follow similar circadian rhythms *in vitro* and *in vivo*

**FIGURE 4:**
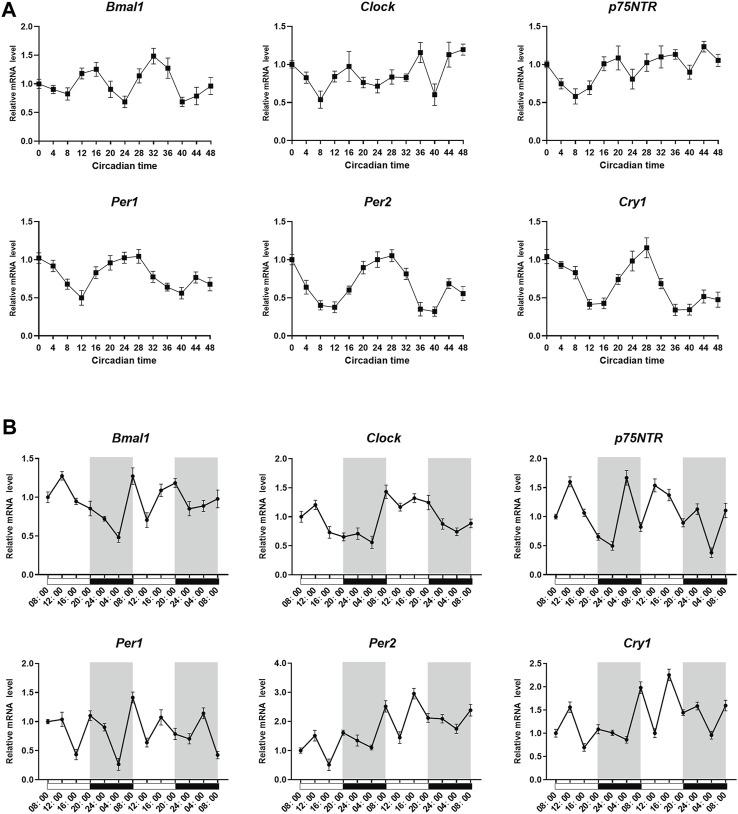
*p75NTR*, *Bmal1*, and *Clock* followed similar circadian rhythms *in vitro* and *in vivo*. **(A)** After synchronization using dexamethasone, ectomesenchymal stem cells (EMSCs) were harvested at the indicated time point and analyzed using qRT-PCR. **(B)** Tooth germs were dissected from postnatal day 1 wildtype mice every 4 h starting at 8:00 a.m. The mRNA expression levels of *Bmal1*, *Clock*, *Per1*, *Per2*, *Cry1*, and *p75NTR* in the tooth germ were analyzed using qRT-PCR at the indicated time points. Data are shown as the means ± standard deviation of three independent experiments (n = 3).

To determine whether p75NTR followed circadian patterns in EMSCs and tooth germ tissues, qRT-PCR was performed at various time points. After synchronization, qRT-PCR analysis revealed that *p75NTR*, *Bmal1*, and *Clock* were expressed at low levels at circadian time 8 (CT8) and high levels at CT16 in EMSCs. High expression of *Per1*, *Per2*, and *Cry1* was observed at CT0 and CT28 (Figure 4A). In tooth germ tissues, qRT-PCR analysis revealed that *p75NTR*, *Bmal1*, and *Clock* were highly expressed at 12:00 p.m. and weakly expressed at approximately 4:00 a.m. (Figure 4B). The expression patterns of *p75NTR* were similar to those of *Bmal1* and *Clock* in EMSCs and tooth germ tissues (Figures 4A, B), which suggests that p75NTR is closely associated with BMAL1 and CLOCK and participates in the regulation of the circadian clock during tooth development and mineralization.

### 3.5 p75NTR positively regulates the expression of BMAL1 and CLOCK

**FIGURE 5:**
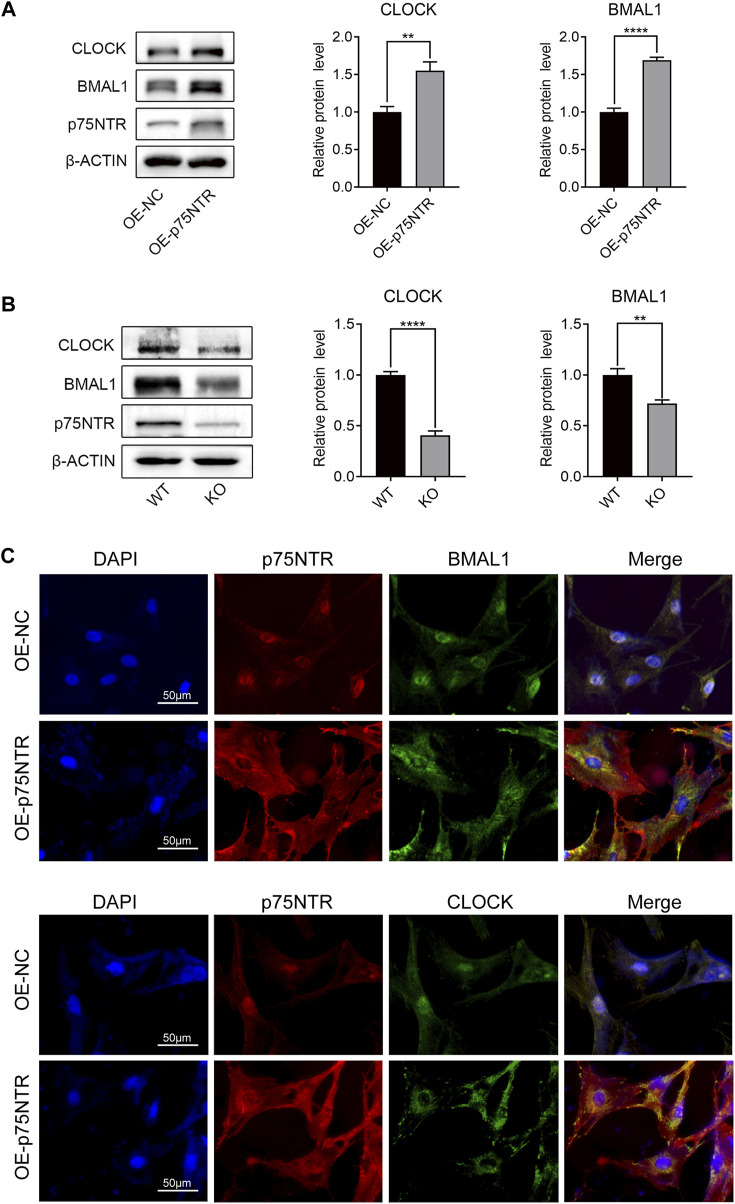
p75NTR positively regulated the expression of BMAL1 and CLOCK. **(A)** Representative western blots and quantitative analysis of BMAL1 and CLOCK proteins in OE-p75NTR and OE-NC ectomesenchymal stem cells (EMSCs) (n = 3). **(B)** Representative Western blot analysis and quantification of BMAL1 and CLOCK in the tooth germs of wildtype (WT) and knockout (KO) mice (n = 3). **(C)** Immunofluorescence staining of p75NTR, BMAL1, and CLOCK in OE-NC and OE-p75NTR EMSCs. Scale bar = 50 μm. Data are shown as mean ± standard deviation. **p ≤ 0.01 and ****p ≤ 0.0001.

BMAL1 and CLOCK are key molecules involved in circadian rhythms. To validate the relationship among p75NTR, BMAL1, and CLOCK, we measured the protein expression levels of BMAL1 and CLOCK in EMSCs overexpressing p75NTR. Western blot analysis revealed that p75NTR increased the expression of BMAL1 and CLOCK (Figure 5A). In contrast, the expression levels of BMAL1 and CLOCK in tooth germ tissues from KO mice were lower than those from WT mice (Figure 5B). The relationship between p75NTR, BMAL1, and CLOCK was confirmed using immunofluorescence. Immunofluorescence analysis showed that in EMSCs overexpressing p75NTR, the expression of BMAL1 and CLOCK increased, with translocation from the nucleus to the cytosol (Figure 5C). Remarkably, overexpression of p75NTR altered cell morphology. Compared with the negative control EMSCs, EMSCs overexpressing p75NTR were polygonal and flat with an enlarged cell body (Figure 5C).

### 3.6 p75NTR positively regulates the odontogenic differentiation and mineralization of EMSCs by upregulating the expression of BMAL1/CLOCK

**FIGURE 6:**
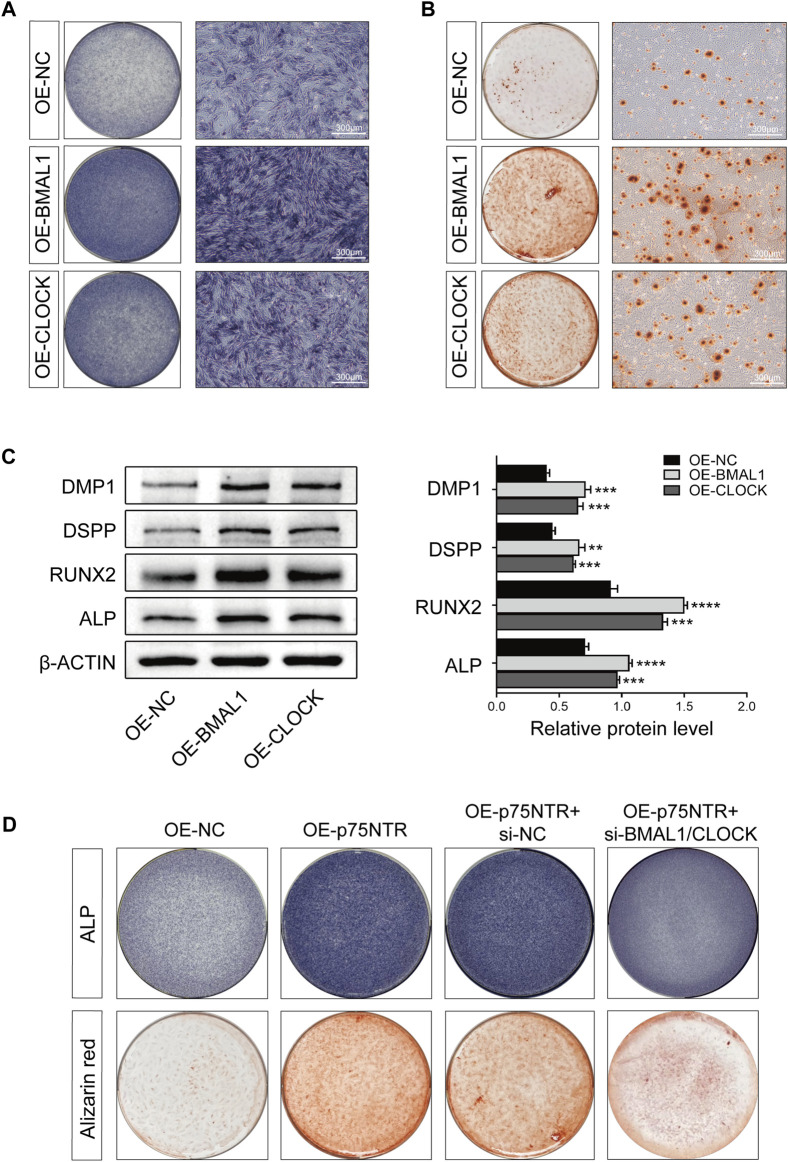
p75NTR positively regulated the odontogenic differentiation and mineralization of EMSCs by upregulating the expression of BMAL1/CLOCK. **(A, B)** EMSCs stably overexpressing BMAL1 or CLOCK (OE-BMAL1 or OE-CLOCK) were generated via lentiviral transduction and treated with the mineralization-inducing medium. **(A)** ALP staining was performed on day 7 and **(B)** alizarin red staining was performed on day 21 (n = 3). Scale bar = 300 μm. **(C)** Representative Western blot and quantification of DMP1, DSPP, RUNX2, and ALP in EMSCs (n = 3). **(D)** EMSCs overexpressing p75NTR were transfected with BMAL1 and CLOCK siRNAs (OE-p75NTR + si-BMAL1/CLOCK) or negative control siRNA (OE-p75NTR + si-NC). These EMSCs were treated with the mineralization-inducing medium. ALP and alizarin red staining were performed on days 7 and 21, respectively (n = 3). Data are shown as mean ± standard deviation. **p ≤ 0.01, ***p ≤ 0.005, and ****p ≤ 0.0001. EMSCs, ectomesenchymal stem cells.

To study the functional consequences of these intermolecular interactions, we first verified the effects of BMAL1 and CLOCK on the odontogenic differentiation of EMSCs. EMSCs overexpressing BMAL1 and CLOCK were used to induce mineralization. ALP staining (Figure 6A) and alizarin red staining (Figure 6B) showed that BMAL1 and CLOCK enhanced ALP activity and the calcium nodule-formation ability of EMSCs. Western blot analysis indicated that BMAL1 and CLOCK promoted the expression of DMP1, DSPP, RUNX2, and ALP in EMSCs (Figure 6C), with BMAL1 having a more significant promoting effect than CLOCK.

Subsequently, we determined whether p75NTR promoted odontogenic differentiation of EMSCs by upregulating the activity of BMAL1/CLOCK. ALP and alizarin red staining revealed that EMSCs overexpressing p75NTR (OE-p75NTR) had significantly enhanced ALP activity and the ability to form calcium nodules compared to the overexpression control EMSCs (OE-NC). p75NTR overexpression EMSCs treated with siRNAs to knockdown BMAL1/CLOCK (OE-p75NTR + si-BMAL1/CLOCK) showed lighter staining than the negative control siRNA EMSCs (OE-p75NTR + si-NC) (Figure 6D).

### 3.7 BMAL1/CLOCK inhibits p75NTR promoter activity and protein expression

**FIGURE 7:**
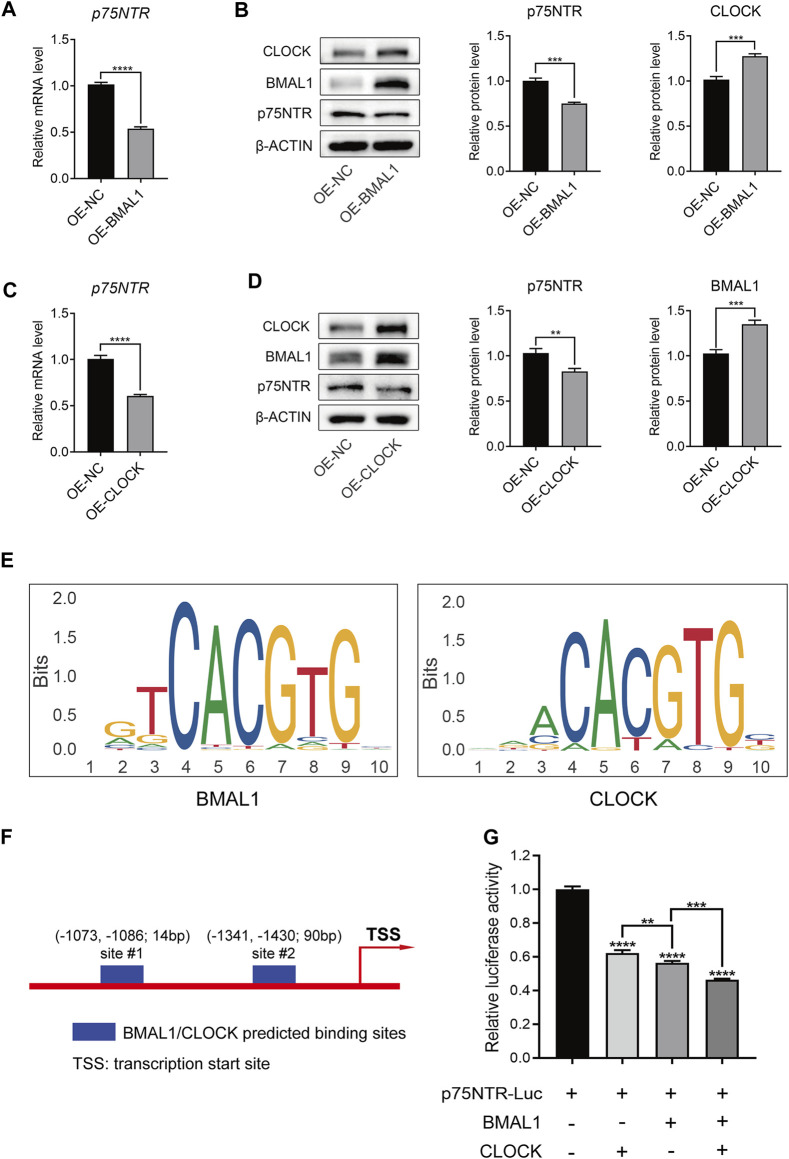
BMAL1/CLOCK inhibited p75NTR promoter activity and protein expression. **(A–D)** The mRNA and protein levels of p75NTR in ectomesenchymal stem cells (EMSCs) overexpressing BMAL1 or CLOCK were detected using **(A and C)** qRT-PCR and **(B and D)** Western blot analyses (n = 3). **(E)** DNA-binding motifs of BMAL1 and CLOCK were obtained from the JASPAR database. **(F)** Predicted BMAL1/CLOCK binding sites in the *p75NTR* promoter region according to the JASPAR database. **(G)** Dual-luciferase reporter assay was performed to measure the activities of the wildtype *p75NTR* promoter in HEK293T cells transfected with BMAL1 expression plasmids and/or CLOCK expression plasmids (n = 3). Data are shown as the mean ± standard deviation. **p ≤ 0.01, ***p ≤ 0.005, and ****p ≤ 0.0001.

To determine whether there is a negative feedback effect between p75NTR and BMAL1/CLOCK, we measured the expression of p75NTR in EMSCs overexpressing BMAL1 and CLOCK. qRT-PCR and Western blot analyses showed that the overexpression of BMAL1 or CLOCK decreased the mRNA and protein levels of p75NTR (Figures 7A–D). Both BMAL1 and CLOCK belong to the bHLH-PAS family and regulate downstream genes by binding to promoters as dimers (Patke et al., 2020a). Figure 7E shows the BMAL1 and CLOCK consensus DNA-binding sequence (–CACGTG–). The JASPAR database further revealed possible binding sites for BMAL1 and CLOCK in the promoter region of p75NTR (Figure 7F). A dual-luciferase reporter gene assay showed that BMAL1 and CLOCK inhibited the expression of p75NTR by inhibiting its promoter activity (Figure 7G); the inhibitory effects of BMAL1 were more pronounced than those of CLOCK.

## 4 Discussion

During tooth development, odontoblasts first form a layer of dentin and retreat to the center of the dental pulp; subsequently, ameloblasts secrete a layer of enamel and retreat to the periphery. These two processes are performed alternately until enamel and dentin are completely formed. This periodic phenomenon observed in the enamel and dentin suggests that the circadian rhythm coordinates tooth formation (Feng et al., 2022). However, the molecular mechanisms by which the circadian clock regulates tooth biomineralization have not been elucidated. In this study, we confirmed that the expression of p75NTR during tooth development follows circadian rhythms and explored the relationship between p75NTR and the core clock factors, BMAL1 and CLOCK, as well as the consequential influence of this relationship on tooth development and mineralization.

p75NTR is involved in various physiological processes and emerging studies have focused on tooth development. As a membrane protein, p75NTR has been used as a surface marker to screen EMSCs derived from the cranial neural crest (Chai et al., 2000). p75NTR + EMSCs are considered progenitors of dental stem cells and provide an optimal stem cell model for studying tooth development (Wen et al., 2012; Wen et al., 2015a). p75NTR activates multiple signaling pathways involved in the regulation of early tooth development (Xing et al., 2016; Li et al., 2017; Wang et al., 2020). Herein, we provide direct evidence that p75NTR regulates tooth biomineralization, as a reduction in tooth mineral density was identified in p75NTR KO mice. Concurrently, the average daily eruption length of the mandibular incisors and the daily mineralization rate of the molars were significantly reduced, suggesting that p75NTR plays a dual role in tooth development by influencing tooth mineralization and circadian rhythm regulation.

Previous studies have proposed that p75NTR functions as a novel clock factor regulated by the core clock factors BMAL1 and CLOCK (Baeza-Raja et al., 2013). p75NTR deficiency disrupts the transcriptional oscillations of clock genes in both the SCN and liver (Baeza-Raja et al., 2013). An important feature of clock genes is their transcriptional oscillation (Zhang et al., 2014a). In this study, the mRNA expression of *p75NTR*, *Bmal1*, and *Clock* showed similar rhythmicity in EMSCs and tooth germ tissues. When mouse tooth germs enter the bell phase, the clock factors (BMAL1, CLOCK, PER1, and PER2) are expressed in odontoblasts and ameloblasts and gradually increase with tooth development (Zheng et al., 2013; Yuan et al., 2022). Histological assessments showed that the expression pattern of p75NTR was similar to that of BMAL1 and CLOCK. Additionally, p75NTR promoted the expression of BMAL1 and CLOCK in EMSCs. These findings suggests that p75NTR is closely associated with the expression of BMAL1 and CLOCK during tooth development. BMAL1 and CLOCK promoted the odontogenic differentiation and mineralization of EMSCs *in vitro*, which is consistent with previous findings (Zheng et al., 2013; Zhou et al., 2018; Xu et al., 2022a). Overexpression of BMAL1 in HAT-7 cells, an ameloblast cell line, increased the expression of amelogenin (Amelx) and kallikrein-related peptidase 4 (Klk4) (Zheng et al., 2013). Dentinogenic differentiation of dental pulp stem cells (DPSCs) can be promoted by BMAL1 via the PI3K/Akt/mTOR pathway (Xu et al., 2022a). Moreover, the absence of BMAL1 expression leads to skeletal mandibular hypoplasia (SMH) in mice (Zhou et al., 2018). Notably, BMAL1 proteins shift from the nucleus to the cytoplasm of cells, followed by circadian patterns corresponding to different cell metabolic activities (Zheng et al., 2013). In EMSCs, shifts in BMAL1 protein localization are regulated by p75NTR. Although many studies supported the important role of BMAL1 in hard tissue formation, BMAL1^−/−^ mice did not show significant changes in the incremental lines of teeth (Papakyrikos et al., 2020). This may be because the circadian rhythm in teeth is not determined by a single molecule but involves a broader regulatory network. BMAL1 and CLOCK usually form BMAL1/CLOCK heterodimers to regulate the transcription of target genes (Patke et al., 2020a). The absence of BMAL1 may be compensated by CLOCK. Coincidentally, BMAL1 and CLOCK were shown to promote each other’s expression in our study; however, few studies have investigated the biological role of CLOCK in tooth development. To the best of our knowledge, this study is the first to demonstrate the direct involvement of CLOCK in the odontogenic differentiation of stem cells.

To further test the involvement of BMAL1/CLOCK in the p75NTR-regulated odontogenic differentiation of EMSCs, siRNAs were used to inhibit the increase in BMAL1 and CLOCK expression in EMSCs overexpressing p75NTR. Blocking BMAL1/CLOCK partially weakened the role of p75NTR in promoting the odontogenic differentiation of EMSCs. Rhythm maintenance depends on negative feedback (Duong et al., 2011a). As expected, BMAL1/CLOCK can inhibit the expression of p75NTR, which may be due to BMAL1/CLOCK reducing the transcriptional activity of p75NTR. This suggests that the p75NTR-BMAL1/CLOCK signaling axis maintains rhythmic mineralization of teeth through its negative feedback regulation. The molecular mechanisms underlying the formation of traditionally described incremental growth lines in dental hard tissues, such as Retzius lines and Incremental lines of von Ebner, hold promise for deciphering through the dynamic thinking of circadian rhythms. The body of evidence has demonstrated that clock factors, including BMAL1, CLCOK, PER1, PER2, and CRY1, can be detected at different stages of tooth development and exhibit oscillations with a 24-h interval (Feng et al., 2022). However, most studies lack direct histological evidence to substantiate the influence of clock factors on these growth lines. The study conducted by Papakyrikos et al. represents the only direct source of evidence, revealing that the absence of the core clock gene *Bmal1* did not result in significant changes in the daily growth lines of mice (Papakyrikos et al., 2020). This suggests the complexity involved in the formation of the incremental growth lines in teeth. Our study provides preliminary validation of the interplay among multiple clock factors in the process of tooth rhythm mineralization. The p75NTR-BMAL1/CLOCK signaling axis may serve as a crucial component in the intricate mechanisms underlying the formation of traditional dentin incremental growth lines. Further histological observations and experimental validation are the focus of our future research endeavors.

## 5 Conclusion

Our study demonstrated that the absence of p75NTR leads to a reduction in tooth mineral density and daily formation, as well as molecular dysregulation during tooth development. p75NTR is a novel clock factor that plays a critical role in the odontogenic differentiation and mineralization of EMSCs through regulation of the p75NTR-BMAL1/CLOCK signaling axis. The associations among and mechanisms of circadian rhythms, tooth development, and biomineralization deserve further exploration.

## Data Availability

The raw data supporting the conclusion of this article will be made available by the authors, without undue reservation.

## References

[B1] Abel J. H. Meeker K. Granados-Fuentes D. St John P. C. Wang T. J. Bales B. B. (2016). Functional network inference of the suprachiasmatic nucleus. Proc. Natl. Acad. Sci. U. S. A. 113, 4512–4517. 10.1073/pnas.1521178113 27044085 PMC4843423

[B2] Baeza-Raja B. Eckel-Mahan K. Zhang L. Vagena E. Tsigelny I. F. Sassone-Corsi P. (2013). p75 neurotrophin receptor is a clock gene that regulates oscillatory components of circadian and metabolic networks. J. Neurosci. 33, 10221–10234. 10.1523/JNEUROSCI.2757-12.2013 23785138 PMC3685830

[B3] Bell-Pedersen D. Cassone V. M. Earnest D. J. Golden S. S. Hardin P. E. Thomas T. L. (2005). Circadian rhythms from multiple oscillators: lessons from diverse organisms. Nat. Rev. Genet. 6, 544–556. 10.1038/nrg1633 15951747 PMC2735866

[B4] Brown S. A. Kowalska E. Dallmann R. (2012a). (Re)inventing the circadian feedback loop. Dev. Cell. 22, 477–487. 10.1016/j.devcel.2012.02.007 22421040

[B5] Buijs F. N. León-Mercado L. Guzmán-Ruiz M. Guerrero-Vargas N. N. Romo-Nava F. Buijs R. M. (2016a). The circadian system: a regulatory feedback network of periphery and brain. Physiol. (Bethesda) 31, 170–181. 10.1152/physiol.00037.2015 27053731

[B6] Chai Y. Jiang X. Ito Y. Bringas P. Han J. Rowitch D. H. (2000). Fate of the mammalian cranial neural crest during tooth and mandibular morphogenesis. Development 127, 1671–1679. 10.1242/dev.127.8.1671 10725243

[B7] Chapple I. L. (1993a). Hypophosphatasia: dental aspects and mode of inheritance. J. Clin. Periodontol. 20, 615–622. 10.1111/j.1600-051x.1993.tb00705.x 8227447

[B8] D’Souza R. N. Aberg T. Gaikwad J. Cavender A. Owen M. Karsenty G. (1999). Cbfa1 is required for epithelial-mesenchymal interactions regulating tooth development in mice. Development 126, 2911–2920. 10.1242/dev.126.13.2911 10357935

[B9] Duong H. A. Robles M. S. Knutti D. Weitz C. J. (2011a). A molecular mechanism for circadian clock negative feedback. Science 332, 1436–1439. 10.1126/science.1196766 21680841 PMC3859310

[B10] Feng G. Zhao J. Peng J. Luo B. Zhang J. Chen L. (2022). Circadian clock-A promising scientific target in oral science. Front. Physiol. 13, 1031519. 10.3389/fphys.2022.1031519 36467684 PMC9708896

[B11] Gekakis N. Staknis D. Nguyen H. B. Davis F. C. Wilsbacher L. D. King D. P. (1998). Role of the CLOCK protein in the mammalian circadian mechanism. Science 280, 1564–1569. 10.1126/science.280.5369.1564 9616112

[B12] Green C. B. Takahashi J. S. Bass J. (2008a). The meter of metabolism. Cell. 134, 728–742. 10.1016/j.cell.2008.08.022 18775307 PMC3760165

[B13] Hasler B. P. Smith L. J. Cousins J. C. Bootzin R. R. (2012a). Circadian rhythms, sleep, and substance abuse. Sleep. Med. Rev. 16, 67–81. 10.1016/j.smrv.2011.03.004 21620743 PMC3177010

[B14] Huang W. Zheng X. Yang M. Li R. Song Y. (2021a). PER2-mediated ameloblast differentiation via PPARγ/AKT1/β-catenin axis. Int. J. Oral Sci. 13, 16. 10.1038/s41368-021-00123-7 34011974 PMC8134554

[B15] Lacruz R. S. Hacia J. G. Bromage T. G. Boyde A. Lei Y. Xu Y. (2012). The circadian clock modulates enamel development. J. Biol. Rhythms. 27, 237–245. 10.1177/0748730412442830 22653892 PMC3511783

[B16] Li G. Liu J. Wang Y. Yang K. Zhao M. Xiao Y. (2017). LNGFR targets the Wnt/β-catenin pathway and promotes the osteogenic differentiation in rat ectomesenchymal stem cells. Sci. Rep. 7, 11021. 10.1038/s41598-017-11555-9 28887537 PMC5591262

[B17] Mohawk J. A. Green C. B. Takahashi J. S. (2012a). Central and peripheral circadian clocks in mammals. Annu. Rev. Neurosci. 35, 445–462. 10.1146/annurev-neuro-060909-153128 22483041 PMC3710582

[B18] Neumann A. M. Schmidt C. X. Brockmann R. M. Oster H. (2019a). Circadian regulation of endocrine systems. Auton. Neurosci. 216, 1–8. 10.1016/j.autneu.2018.10.001 30598120

[B19] Ohtsuka M. Saeki S. Igarashi K. Shinoda H. (1998a). Circadian rhythms in the incorporation and secretion of 3H-proline by odontoblasts in relation to incremental lines in rat dentin. J. Dent. Res. 77, 1889–1895. 10.1177/00220345980770110501 9823727

[B20] Ohtsuka-Isoya M. Hayashi H. Shinoda H. (2001a). Effect of suprachiasmatic nucleus lesion on circadian dentin increment in rats. Am. J. Physiol. Regul. Integr. Comp. Physiol. 280, R1364–R1370. 10.1152/ajpregu.2001.280.5.R1364 11294755

[B21] Papakyrikos A. M. Arora M. Austin C. Boughner J. C. Capellini T. D. Dingwall H. L. (2020). Biological clocks and incremental growth line formation in dentine. J. Anat. 237, 367–378. 10.1111/joa.13198 32266720 PMC7369199

[B22] Patke A. Young M. W. Axelrod S. (2020a). Molecular mechanisms and physiological importance of circadian rhythms. Nat. Rev. Mol. Cell. Biol. 21, 67–84. 10.1038/s41580-019-0179-2 31768006

[B23] Reppert S. M. Weaver D. R. (2001a). Molecular analysis of mammalian circadian rhythms. Annu. Rev. Physiol. 63, 647–676. 10.1146/annurev.physiol.63.1.647 11181971

[B24] Ritchie H. (2018a). The Functional Significance of dentin sialoprotein-phosphophoryn and dentin sialoprotein. Int. J. Oral Sci. 10, 31. 10.1038/s41368-018-0035-9 30393383 PMC6215839

[B25] Smith T. M. (2006a). Experimental determination of the periodicity of incremental features in enamel. J. Anat. 208, 99–113. 10.1111/j.1469-7580.2006.00499.x 16420383 PMC2100182

[B26] Tang Q. Xie M. Yu S. Zhou X. Xie Y. Chen G. (2019). Periodic oxaliplatin administration in synergy with PER2-mediated PCNA transcription repression promotes chronochemotherapeutic efficacy of OSCC. Adv. Sci. (Weinh). 6, 1900667. 10.1002/advs.201900667 31728273 PMC6839751

[B27] Wang Y. Yang K. Li G. Liu R. Liu J. Li J. (2020). p75NTR-/- mice exhibit an alveolar bone loss phenotype and inhibited PI3K/Akt/β-catenin pathway. Cell. Prolif. 53, e12800. 10.1111/cpr.12800 32215984 PMC7162804

[B28] Wen X. Liu L. Deng M. Liu R. Zhang L. Nie X. (2015a). *In vitro* cementoblast-like differentiation of postmigratory neural crest-derived p75(+) stem cells with dental follicle cell conditioned medium. Exp. Cell. Res. 337, 76–86. 10.1016/j.yexcr.2015.07.001 26165934

[B29] Wen X. Liu L. Deng M. Zhang L. Liu R. Xing Y. (2012). Characterization of p75(+) ectomesenchymal stem cells from rat embryonic facial process tissue. Biochem. Biophys. Res. Commun. 427, 5–10. 10.1016/j.bbrc.2012.08.109 22982680

[B30] Xing Y. Nie X. Chen G. Wen X. Li G. Zhou X. (2016). Comparison of P75 NTR-positive and -Negative etcomesenchymal stem cell odontogenic differentiation through epithelial-mesenchymal interaction. Cell. Prolif. 49, 185–194. 10.1111/cpr.12248 27038014 PMC6496417

[B31] Xu H. Zhao J. Chen G. Yuan Z. Liu J. (2022a). Effects of BMAL1 on dentinogenic differentiation of dental pulp stem cells via PI3K/Akt/mTOR pathway. Int. Endod. J. 55, 505–516. 10.1111/iej.13720 35263812

[B32] Ye L. MacDougall M. Zhang S. Xie Y. Zhang J. Li Z. (2004). Deletion of dentin matrix Protein-1 leads to a partial failure of maturation of predentin into dentin, hypomineralization, and expanded cavities of pulp and root canal during postnatal tooth development. J. Biol. Chem. 279, 19141–19148. 10.1074/jbc.M400490200 14966118

[B33] Yu T. Klein O. D. (2020a). Molecular and cellular mechanisms of tooth development, homeostasis and repair. Development 147, dev184754. 10.1242/dev.184754 31980484 PMC6983727

[B34] Yuan H. Xie B. Yu X. Lin C. Li M. Zhang Y. (2022). A potential role of p75NTR in the regulation of circadian rhythm and incremental growth lines during tooth development. Front. Physiol. 13, 981311. 10.3389/fphys.2022.981311 36213234 PMC9539461

[B35] Zhang R. Lahens N. F. Ballance H. I. Hughes M. E. Hogenesch J. B. (2014a). A circadian gene expression atlas in mammals: implications for biology and medicine. Proc. Natl. Acad. Sci. U. S. A. 111, 16219–16224. 10.1073/pnas.1408886111 25349387 PMC4234565

[B36] Zhao M. Wang Y. Li G. Li J. Yang K. Liu C. (2020). The role and potential mechanism of p75NTR in mineralization via *in vivo* p75NTR knockout mice and *in vitro* ectomesenchymal stem cells. Cell. Prolif. 53, e12758. 10.1111/cpr.12758 31922317 PMC7048213

[B37] Zhao M. Wen X. Li G. Ju Y. Wang Y. Zhou Z. (2019). The spatiotemporal expression and mineralization regulation of p75 neurotrophin receptor in the early tooth development. Cell. Prolif. 52, e12523. 10.1111/cpr.12523 30357966 PMC6430448

[B38] Zheng L. Papagerakis S. Schnell S. D. Hoogerwerf W. A. Papagerakis P. (2011a). Expression of clock proteins in developing tooth. Gene Expr. Patterns. 11, 202–206. 10.1016/j.gep.2010.12.002 21156215 PMC3073654

[B39] Zheng L. Seon Y. J. Mourão M. A. Schnell S. Kim D. Harada H. (2013). Circadian rhythms regulate amelogenesis. Bone 55, 158–165. 10.1016/j.bone.2013.02.011 23486183 PMC3650122

[B40] Zhou X. Yu R. Long Y. Zhao J. Yu S. Tang Q. (2018). BMAL1 deficiency promotes skeletal mandibular hypoplasia via OPG downregulation. Cell. Prolif. 51, e12470. 10.1111/cpr.12470 30117209 PMC6528896

