# Supplementary figures and images for: p75NTR promotes tooth rhythmic mineralization via upregulation of BMAL1/CLOCK
Source: Front Cell Dev Biol. 2023 Nov 7;11:1283878. doi: 10.3389/fcell.2023.1283878 (PMC10662321; doi:10.3389/fcell.2023.1283878)

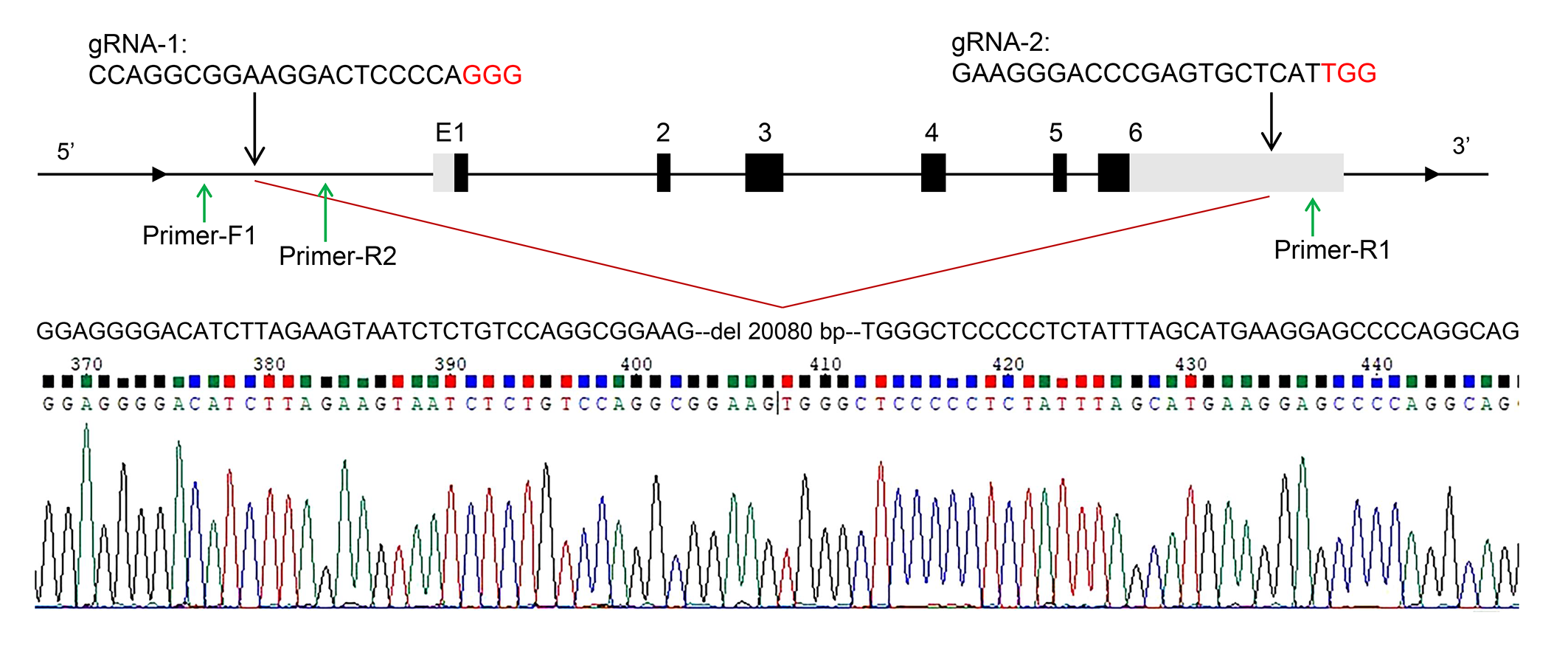

Supplement: Supplementary file 1 [file Image1.TIF]
