# Supplementary material for: p75NTR promotes tooth rhythmic mineralization via upregulation of BMAL1/CLOCK
Source: Front Cell Dev Biol. 2023 Nov 7;11:1283878. doi: 10.3389/fcell.2023.1283878 (PMC10662321; doi:10.3389/fcell.2023.1283878)
Supplement: Supplementary file 2 [file DataSheet1.DOCX]

Supplementary Material

## Supplementary Figures


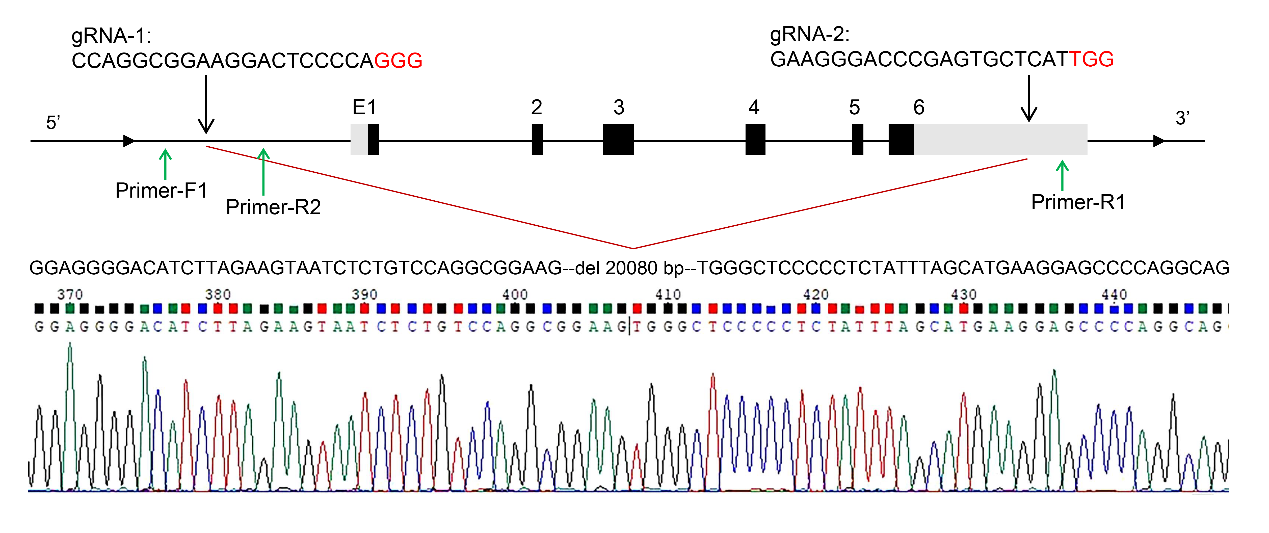


**Supplementary Figure 1.** Generation of p75NTR knockout mice using the CRISPR/Cas9 system. Two gRNAs (gRNA-1 and gRNA-2) were designed to delete exons 1–6 of the *p75NTR* gene. Primer-F1 and Primer-R1 were used to amplify the knockout allele, whereas Primer-F1 and Primer-R2 were used to amplify the wild-type allele. Deletion of p75NTR was confirmed via sequencing.
